# Genetic Contributors of Incident Stroke in 10,700 African Americans With Hypertension: A Meta-Analysis From the Genetics of Hypertension Associated Treatments and Reasons for Geographic and Racial Differences in Stroke Studies

**DOI:** 10.3389/fgene.2021.781451

**Published:** 2021-12-21

**Authors:** Nicole D. Armstrong, Vinodh Srinivasasainagendra, Amit Patki, Rikki M. Tanner, Bertha A. Hidalgo, Hemant K. Tiwari, Nita A. Limdi, Ethan M. Lange, Leslie A. Lange, Donna K. Arnett, Marguerite R. Irvin

**Affiliations:** ^1^ Department of Epidemiology, University of Alabama at Birmingham, Birmingham, AL, United States; ^2^ Department of Biostatistics, University of Alabama at Birmingham, Birmingham, AL, United States; ^3^ Department of Neurology, University of Alabama at Birmingham, Birmingham, AL, United States; ^4^ Division of Biomedical Informatics and Personalized Medicine, Department of Medicine, University of Colorado Anschutz Medical Campus, Aurora, CO, United States; ^5^ College of Public Health, University of Kentucky, Lexington, KY, United States

**Keywords:** incident stroke, hypertension, antihypertensives, disparities, polygenic risk score, genome wide association studies

## Abstract

**Background:** African Americans (AAs) suffer a higher stroke burden due to hypertension. Identifying genetic contributors to stroke among AAs with hypertension is critical to understanding the genetic basis of the disease, as well as detecting at-risk individuals.

**Methods:** In a population comprising over 10,700 AAs treated for hypertension from the Genetics of Hypertension Associated Treatments (GenHAT) and Reasons for Geographic and Racial Differences in Stroke (REGARDS) studies, we performed an inverse variance-weighted meta-analysis of incident stroke. Additionally, we tested the predictive accuracy of a polygenic risk score (PRS) derived from a European ancestral population in both GenHAT and REGARDS AAs aiming to evaluate cross-ethnic performance.

**Results:** We identified 10 statistically significant (*p* < 5.00E-08) and 90 additional suggestive (*p* < 1.00E-06) variants associated with incident stroke in the meta-analysis. Six of the top 10 variants were located in an intergenic region on chromosome 18 (*LINC01443-LOC644669*). Additional variants of interest were located in or near the *COL12A1, SNTG1*, *PCDH7*, *TMTC1*, and *NTM* genes*.* Replication was conducted in the Warfarin Pharmacogenomics Cohort (WPC), and while none of the variants were directly validated, seven intronic variants of *NTM* proximal to our target variants, had a *p*-value <5.00E-04 in the WPC. The inclusion of the PRS did not improve the prediction accuracy compared to a reference model adjusting for age, sex, and genetic ancestry in either study and had lower predictive accuracy compared to models accounting for established stroke risk factors. These results demonstrate the necessity for PRS derivation in AAs, particularly for diseases that affect AAs disproportionately.

**Conclusion:** This study highlights biologically plausible genetic determinants for incident stroke in hypertensive AAs. Ultimately, a better understanding of genetic risk factors for stroke in AAs may give new insight into stroke burden and potential clinical tools for those among the highest at risk.

## Introduction

As the second-leading global cause of death and a leading cause of disability-adjusted life-years lost (Katan and Luft, 2018), stroke is a major public health burden especially among African Americans (AAs) who have a 50% higher risk of stroke (Howard et al., 2011). The role of genetics on stroke risk has been evidenced through twin studies, where monozygotic twins are more likely to be concordant than dizygotic twins (odds ratio for concordance (OR): 1.65, 95% CI: 1.2–2.3) (Flossmann et al., 2004). Previously reported heritability estimates of stroke based on data from genome-wide association studies (GWAS) are comparable for AAs and individuals of European Ancestry (EAs); 38% for EAs (Bevan et al., 2012) and 35% for AAs (Traylor et al., 2017), yet AAs contribute far less data to a large body of literature on stroke genetic risk factors (Bentley et al., 2020).

GWAS of stroke in European populations have identified susceptibility loci located on chromosomes 4q25 and 9p21 (Gretarsdottir et al., 2008), 7p21.1 (International Stroke Genetics, 2012), 6p21 (Holliday et al., 2012), 12p13 (Ikram et al., 2009), 12q24 (Kilarski et al., 2014), and 16q22 (Traylor et al., 2012). Overall, there has been a lack of similar primary GWAS discovery efforts in AAs, and results from EAs have not generally replicated in AAs (Carty et al., 2015; Peprah et al., 2015). For example, of 520,000 participants in the large trans-ethnic GWAS meta-analysis from the MEGASTROKE consortium, only ∼4% of participants were AAs (Malik et al., 2018). The only other study with a large number of AAs, the Consortium of Minority Population Genome-Wide Association Studies of Stroke (COMPASS), confirmed the need for race-specific stroke discovery, finding weak or no validation across ethnic groups (Carty et al., 2015; Keene et al., 2020). Given the relative lack of data on this race group in the published literature, additional stroke variant discovery in this population remains needed.

GWAS data capitalizes on the polygenic nature of common diseases and has been collected on a large scale to provide useful health information over traditional clinical risk factors. So far GWAS data at the single variant level, even from large consortia studies such as those described above, has been difficult to translate to the clinic. More recently, cumulative single variant effects from GWAS, at thousands to millions of variants, are being used to estimate polygenic risk scores (PRS), which may prove useful for stroke risk prediction and prevention. Unfortunately, few stroke PRS have been developed in populations including AAs and, to the best of our knowledge, only one PRS was developed exclusively in individuals of African descent, consisting of only 29 variants (Traylor et al., 2017).

Abraham et al. (2019) comprised a composite meta-risk PRS (metaPRS) for ischemic stroke leveraging publicly available GWAS data for stroke and 19 stroke-related phenotypes generated from the British European dataset of the United Kingdom Biobank (UKB, *n* = 407,388). Under a split sample design, participants with ‘any stroke’ event were oversampled for the derivation set (*n* = 11,995 total; *n =* 2,065 with “any stroke” events) followed by validation in the remaining ∼395,000 participants. The authors did not report on score validation in the small sample of available individuals of African descent from the UKB (N∼8,000). Given difficulties in cross-ethnic validation of stroke variants in GWAS, we suspected that the publicly available Abraham composite metaPRS will not perform adequately in AAs.

The goals of the current study, which we set among AAs at elevated risk for stroke due to hypertension (HTN), were two-pronged. First, we aimed to increase stroke GWAS data available in this race group using data from the Genetics of Hypertension Associated Treatments (GenHAT) study and the Reasons for Geographic and Racial Differences in Stroke (REGARDS) studies. Second, we aimed to assess the predictive ability of the UKB derived stroke PRS (Abraham et al., 2019) in these two populations given the lack of previous cross-ethnic validation.

## Materials and Methods

### Study Participants

GenHAT was an ancillary pharmacogenetic study to the Antihypertensive and Lipid-Lowering Treatment To Prevent Heart Attack Trial (ALLHAT) and REGARDS is an ongoing cohort study of stroke risk in the continental US. The GenHAT and REGARDS studies contributed genetic and phenotypic data on 10,717 AAs with HTN at baseline (GenHAT *n* = 6,908; REGARDS *n* = 3,809), further detailed in the Supplementary Material. Participants in GenHAT were randomized to either chlorthalidone, a thiazide diuretic (TD), or lisinopril, an angiotensin-converting enzyme (ACE) inhibitor, while REGARDS participants were taking a TD, ACE inhibitor, or a combination of both for inclusion in the analysis (Supplementary Table S1). All studies were approved by local institutional review boards and/or ethics committees. All participants provided written informed consent.

### Definition of Incident Stroke

In GenHAT, incident stroke was defined as the rapid onset of persistent neurologic deficit attributable to an obstruction or rupture of the arterial system, including stroke occurring during surgery, not known to be secondary to brain trauma, tumor, infection, or other non-ischemic cause and must last more than 24 h unless death supervenes or there is a demonstrable lesion compatible with acute stroke on CT or MRI scan (ALLHAT Protocol, 2000). In REGARDS, any suspected stroke events were identified every 6 months *via* telephone interview. The medical records associated with these events were retrieved and adjudicated by a physician, using the World Health Organization definition of stroke as focal neurologic symptoms lasting more than 24 h or those with neuroimaging data consistent with stroke (WHO Task Force on Stroke and other Cerebrovascular Disorders, 1989; Howard et al., 2011). For REGARDS, all stroke events occurring before or on September 30, 2019 were included in this analysis. REGARDS participants with a history of previous stroke were excluded.

### Genotyping, Quality Control, and Imputation

Genome-wide genotyping was performed within each study independently using Illumina Infinium Multi-Ethnic AMR/AFR Extended BeadChip arrays (MEGA chip; Illumina, Inc., San Diego, CA). Supplementary Table S2 includes detailed information on the variant and sample quality control (QC). Filtered genotype calls were imputed using the NHLBI Trans-omics for Precision Medicine (TOPMed) release 2 (Freeze 8) reference panel, which leverages data on ∼186,000 samples (∼30% AA) (Fuchsberger et al., 2015; Das et al., 2016; Taliun et al., 2021). Post-imputation QC excluded variants with imputation quality scores (Rsq) < 0.3 and a Minor Allele Count (MAC) < 20 in each cohort, as previously described using TOPMed data (Sarnowski et al., 2021). The variants not represented in both GenHAT and REGARDS were excluded from subsequent analyses.

### Statistical Analysis

Baseline descriptive statistics for cases and controls are presented as counts (percentages) for categorical variables or mean ± standard deviation (SD) for continuous variables, and were compared using *χ* ^2^ tests and *t*-tests, respectively (Table 1).

**TABLE 1:** Demographic characteristics of GenHAT and REGARDS participants stratified by incident stroke case-control status.

	GenHAT			REGARDS		
	Cases (*n* = 366)	Controls (*n* = 6,542)	*p*	Cases (*n* = 280)	Controls (*n* = 3,529)	*p*
Age, years	68.70 ± 8.19	66.02 ± 7.70	<0.001	67.41 ± 9.01	64.42 ± 8.81	<0.001
Sex						
Male	191 (52.2%)	2,893 (44.2%)	0.003	102 (36.4%)	1,310 (37.1%)	0.868
Female	175 (47.8%)	3,649 (55.8%)		178 (63.6%)	2,219 (62.9%)	
Antihypertensive class						
Thiazide diuretic	190 (51.9%)	4,107 (62.8%)	<0.001	88 (31.4%)	1,418 (40.2%)	0.013
ACE Inhibitor	176 (48.1%)	2,435 (37.2%)		123 (43.9%)	1,311 (37.1%)	
Combination therapy^a^	NA	NA		69 (24.6%)	800 (22.7%)	
Cigarette smoking	89 (31.7%)	1,506 (27.1%)	0.104	39 (14.0%)	591 (16.8%)	0.255
DM	188 (51.4%)	2,588 (39.6%)	<0.001	136 (49.5%)	1,325 (38.2%)	<0.001
BMI	29.65 ± 6.34	30.52 ± 6.60	0.014	31.20 ± 5.81	31.83 ± 6.76	0.126
SBP, mmHg	148.37 ± 15.98	146.13 ± 15.75	0.008	135.65 ± 16.90	132.03 ± 16.78	0.001
DBP, mmHg	84.64 ± 10.90	84.88 ± 10.12	0.667	78.42 ± 10.41	78.74 ± 10.04	0.603
eGFR, mL/min/1.73 m^2^	78.62 ± 22.88	82.95 ± 21.68	<0.001	83.44 ± 25.52	86.47 ± 27.59	0.079

a GenHAT participants were taking either thiazide diuretic or ACE inhibitor at baseline.

Categorical variables are described as N (%), while continuous variables are described as mean ± standard deviation. Abbreviations: TD, thiazide diuretic class; ACE, angiotensin-converting enzyme; DM, diabetes mellitus; BMI, body mass index; SBP, systolic blood pressure; DBP, diastolic blood pressure; eGFR, estimated glomerular filtration rate.

Firth logistic regression models implemented in PLINK2 (Ma et al., 2013; Chang et al., 2015) were used for genome-wide association analysis of incident stroke status. Models of the imputed effect allele dosage were adjusted for age, sex, and the top 10 principal components (PCs) for ancestry derived in EIGENSTRATv6.1.4 (Price et al., 2006). An inverse variance-weighted, fixed effects meta-analysis was performed on the summary statistics from GenHAT and REGARDS, using METAL software (Willer et al., 2010). Statistical heterogeneity was evaluated using Cochran’s chi-square test in METAL. Regional plots were created using LocusZoom v0.12 (Pruim et al., 2010; Boughton et al., 2021). Gene annotation was completed using ANNOVAR (Wang et al., 2010). Genome-wide significance was set at *p* < 5.00E-08 after a Bonferroni correction for multiple testing. A randomization drug-adjusted sensitivity model was performed in the GenHAT data to account for any effects of the antihypertensive agent, and the results were similar to the discovery model (data not shown). To address potential issues associated with case-control imbalance, we ran sensitivity analyses in the GenHAT and REGARDS data using the saddle point approximation (SPA) in the Scalable and Accurate Implementation of Generalized (SAIGE) program and meta-analyzed the results (Zhou et al., 2018). This approach provides good control of type 1 error for binary traits, however the SPA approach in SAIGE is very conservative and has been described to result in inflated effect-size estimates (Mbatchou et al., 2021). To determine if there are any shared genetic risk factors for stroke with EAs, we conducted a fixed effects, inverse-variance weighted meta-analysis incorporating top (*p* < 1.00E-07) variants (*n* = 356) from the publicly available MEGASTROKE European analysis summary statistics (Malik et al., 2018).

To analyze the putative biological mechanisms of a subset of significant and suggestive variants identified in the meta-analysis with *p* < 1.00E-06, we utilized the Functional Mapping and Annotation of Genome-Wide Association Studies (FUMA) version 1.3.6a online platform and the GENE2FUNC process (Watanabe et al., 2017). Within FUMA, over 22,000 genes underwent zero-mean normalization and log2 transformation of the expression values [zero mean of log2 (reads per kilobase per million +1)]. Differentially expressed gene (DEG) sets for each of the 53 specific tissue types from the Genotype-Tissue Expression (GTEx) project version eight RNA sequencing data (GTEx Consortium, 2017), were determined from two-sided Student’s *t*-test performed per gene per tissue type against all other tissue types. Genes with *p* ≤ 0.05 after Bonferroni correction and absolute log fold change of ≥0.58, were included in the DEG set for a given tissue (Watanabe et al., 2017). Furthermore, FUMA distinguished between genes that were upregulated and downregulated in a specific tissue type compared to other tissues *via* the sign of the t-statistic (Watanabe et al., 2017). Genes identified from the meta-analysis were uploaded into FUMA and mapped to Ensembl identifiers. Our prioritized genes were tested against biologically relevant tissue (brain *n* = 12, artery *n* = 3, heart *n* = 2, and kidney *n* = 2) DEG sets using hypergeometric tests to evaluate if the targeted genes were overrepresented in FUMA DEG sets in specific tissue types. Multiple testing correction was performed using a Benjamini-Hochberg adjustment. Statistical significance was calculated using a p-threshold of *p* < 0.05.

We utilized the publicly available risk score weights for the Abraham metaPRS to assess the predictive accuracy of this score in both GenHAT and REGARDS AAs (Abraham et al., 2019). Specifically, the PRS were generated separately for each study population using the allelic scoring option in PLINK2, resulting in the inclusion of 466,657 and 466,614 variants in GenHAT and REGARDS, respectively. We then employed a series of nine logistic regression models. Model 1 (reference model) regressed the ‘any stroke’ outcome on age, sex, and PCs 1–10. The metaPRS and clinical risk factors (SBP, DBP, DM, baseline cigarette smoking, or BMI) were individually added to Model 1. A clinical model added each clinical risk factor (SBP + DBP + DM + baseline cigarette smoking + BMI) to model 1. Lastly, we generated a composite model that consisted of Model one plus clinical factors and the metaPRS (SBP + DBP + DM + baseline cigarette smoking + BMI + metaPRS). Using the predicted values for stroke, the performance of each model was determined by the area under the receiver operator characteristic (ROC) curve (AUC). Delong’s test for two correlated ROC curves compared the pairwise performance of the nested models (i.e., model one to each of the subsequent models). The fit of each model was determined by Nagelkerke’s pseudo-R^2^. All predictive regression modeling and model fit calculations were performed in R version 3.6.2.

### Replication for Meta-analysis

We sought replication in AAs from the University of Alabama at Birmingham Warfarin Pharmacogenomics Cohort (WPC) (Shendre et al., 2016; Yanik et al., 2017). The WPC replication population included 790 AAs, of which 145 had an incident stroke. In the WPC, incident stroke case-control status was regressed onto genotypes imputed to the TOPMed release 2 (Freeze 8) reference panel, adjusting for age, sex, the top 4 PCs, and genotyping platform using PLINK2. Further details are described in the Supplementary Material. We also expanded our replication lookups to the region around our index variants from the meta-analysis. Based on prior research, we considered a window within 100 KB of the target variant (Genomes Project et al., 2015).

Further replication was obtained using the publicly available summary statistics from the recent COMPASS meta-analysis study (Keene et al., 2020). In the COMPASS meta-analysis, 22,051 AAs were included, of which 3,734 had a physician-adjudicated stroke. Due to the imputation reference panel differences (TOPMed vs. 1,000 Genomes), we did not have sufficient overlap to replicate on the variant level. Our replication efforts examined all variants located in the gene region [5′ untranslated region (UTR) through the 3′ UTR] of the target variant, or in the case of intergenic variants, the entire region between the two flanking genes. Of note, there is an overlap of 864 participants (66 cases) from our discovery REGARDS population and those included in the published COMPASS meta-analysis of IS, which could inflate the results on a single-variant level.

## Results

The baseline characteristics for GenHAT and REGARDS participants are presented in Table 1. In the 6,908 GenHAT participants, approximately 5% (*n* = 366) had an incident stroke. Males comprised approximately 52% of stroke cases and 44% of controls. The average age of cases was nearly 69 years of age, while the controls were younger at 66 years (*p* < 0.001). In GenHAT, stroke cases were more likely current cigarette smokers (32% v. 27%; *p* = 0.104) and diabetic (51% v. 40%; *p* < 0.001) compared to controls. Likewise, stroke cases had worse kidney function as estimated by the mean glomerular filtration rate (eGFR) (78.62 v. 82.95 ml/min/1.73 m^2^; *p* < 0.001), higher mean SBP (148.37 v. 146.13 mmHg; *p* = 0.008) and a negligible difference in mean DBP (84.64 v. 84.88 mmHg; *p* = 0.667) compared to controls.

In the 3,809 REGARDS participants, approximately 7% (*n* = 280) had an incident stroke. Males comprised approximately 36% of incident stroke cases versus 37% of controls. The average age of stroke cases was 67 vs. 64 years for controls (*p* < 0.001). Of stroke cases, 14% were current cigarette smokers compared to 17% of controls (*p* = 0.255), and almost 50% had DM versus 38% of controls (*p* < 0.001). The stroke cases had slightly higher baseline SBP (135.65 mmHg v. 132.03 mmHg; *p* = 0.001), lower baseline DBP (78.42 mmHg v. 78.74 mmHg; *p* = 0.603), and worse kidney function (83.44 ml/min/1.73 m^2^ v. 86.47 ml/min/1.73 m^2^; *p* = 0.079) compared to controls (Table 1).

**FIGURE 1:**
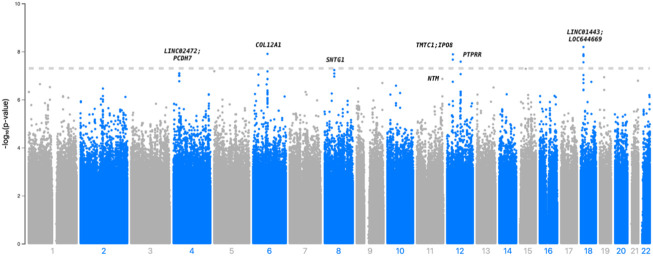
Manhattan plot depicting the top associations with incident stroke in GenHAT-REGARDS meta-analysis. The dotted line is representative of genome-wide significance (*p* < 5.00E-08).

In Table 2, we present 21 top variants (*p* < 1.00E-07) across nine unique genes, of which 10 variants were statistically significant (*p* < 5.00E-08) from the inverse variance-weighted meta-analysis (Figure 1). An additional 79 variants were marginally significant (*p* < 1.00E-06) and are described in Supplementary Table S3. In the sensitivity analysis using SAIGE, we observed consistent results in our top finding, although we saw marginal reduction in the significance (Supplementary Table S4). The most significant variant, rs117880209 (*p* = 6.45E-09), was located in the intergenic region between *LINC01443* and *LOC644669* on chromosome 18. At this variant the odds ratio for incident stroke was 2.98 (95% CI 2.06-4.31) for the C (v. T) allele and the direction of the effect was consistent across both GenHAT and REGARDS. An additional 10 variants in this intergenic region met or exceeded *p* < 1.00E-06.

**TABLE 2:** Top variants (*p* < 1.00E-07) associated with incident stroke from inverse variance-weighted meta-analysis.

rsID	CHR	BP (hg38)	A1/A2	EAF	OR	95% CI	^a^ *p*	^b^Direction	Location	Gene(s)
rs117880209	18	14,996,132	C/T	0.012	2.98	2.06, 4.31	6.45E-09	−−	intergenic	*LINC01443; LOC644669*
rs144162260	6	75,188,146	G/T	0.005	4.32	2.61, 7.15	1.25E-08	−−	intronic	*COL12A1*
rs117791256	18	15,006,517	C/G	0.011	2.95	2.03, 4.28	1.31E-08	++	intergenic	*LINC01443; LOC644669*
rs536017124	12	30,539,571	A/C	0.004	4.79	2.79, 8.22	1.32E-08	++	intergenic	*TMTC1; IPO8*
rs142422295	18	15,015,740	C/T	0.011	2.93	2.02, 4.25	1.51E-08	−−	intergenic	*LINC01443; LOC644669*
rs145341988	18	15,015,569	T/C	0.011	2.93	2.02, 4.25	1.51E-08	++	intergenic	*LINC01443; LOC644669*
rs140550089	18	15,003,860	C/T	0.011	2.93	2.02, 4.25	1.51E-08	−−	intergenic	*LINC01443; LOC644669*
rs192840029	12	30,513,818	A/G	0.004	4.67	2.72, 8.00	2.17E-08	++	intergenic	*TMTC1; IPO8*
rs568505299	12	70,890,293	T/C	0.005	3.95	2.44, 6.41	2.64E-08	++	intronic	*PTPRR*
rs58633304	18	14,988,000	C/A	0.013	2.76	1.93, 3.96	2.79E-08	−−	intergenic	*LINC01443; LOC644669*
rs186234470	15	48,470,292	T/C	0.011	2.77	1.92, 4.00	5.20E-08	++	intronic	*FBN1*
rs117962542	8	50,357,386	A/G	0.003	5.59	3.00, 10.41	5.91E-08	++	intronic	*SNTG1*
rs116671900	5	1,833,726	T/C	0.002	7.60	3.64, 15.87	6.59E-08	++	intergenic	*NDUFS6; LINC02116*
rs145817478	6	75,173,029	G/A	0.004	4.27	2.52, 7.23	6.70E-08	−−	intronic	*COL12A1*
rs74599173	4	30,537,733	A/G	0.004	4.48	2.59, 7.74	8.09E-08	++	intergenic	*LINC02472; PCDH7*
rs77853510	4	30,553,234	A/G	0.004	4.48	2.59, 7.74	8.09E-08	++	intergenic	*LINC02472; PCDH7*
rs118141576	8	50,326,925	A/T	0.003	5.09	2.81, 9.22	8.18E-08	++	intronic	*SNTG1*
rs541454296	12	70,806,203	C/T	0.003	5.52	2.95, 10.31	8.72E-08	−−	intronic	*PTPRR*
rs143089250	6	28,694,541	C/T	0.003	5.79	3.04, 11.04	9.02E-08	−−	intergenic	*LINC00533; LINC01623*
rs117306,905	18	15,004,526	T/C	0.013	2.67	1.86, 3.84	9.50E-08	++	intergenic	*LINC01443; LOC644669*
rs75989184	4	30,534,014	G/C	0.004	4.42	2.56, 7.63	9.93E-08	−−	intergenic	*LINC02472; PCDH7*

a Genome-wide statistical significance after multiple testing correction, *p* < 5.00e-08.

b Direction order: GenHAT, REGARDS.

Abbreviations: rsID, reference SNP cluster ID; CHR, chromosome number; BP, base position from hg38; A1, effect allele; A2, allele 2; EAF, effect allele frequency; OR, odds ratio; CI, confidence interval.

Furthermore, we observed 13 intronic variants in *COL12A1* on chromosome 6 with *p* < 1.00E-6. One intronic variant in this gene, rs144162260, reached statistical significance (*p* = 1.25E-08; OR = 4.32; 95% CI = 2.61–7.15). Three additional variants exceeded genome-wide significance, including two intergenic variants between *TMTC1* and *IP O 8* (top variant: rs536017124; *p* = 1.32E-08; OR = 4.79; 95% CI = 2.79–8.22), and one intronic variant of *PTPRR* (rs568505299; *p* = 2.64E-08, OR = 3.95, 95% CI = 2.44–6.41) (Table 2).

Other variants of biological interest include 11 intergenic variants between *LINC02472* and *PCDH7* (top variant: rs74599173, *p* = 8.09E-08), three intronic variants of *SNTG1* (top variant: rs117962542, *p* = 5.91E-08), and two intronic variants of *NTM* (top variant: rs185159493, *p* = 1.37E-07) (Table 2; Supplementary Table S3). The genomic inflation factor (*λ*) from the individual cohorts (GenHAT *λ* = 0.952; REGARDS *λ* = 1.002) and the meta-analysis (*λ* = 0.992) showed no evidence of systematic inflation (Supplementary Table S5; Supplementary Figure S1). LocusZoom plots of the top variants in *COL12A1, PTPRR, NTM,* and the intergenic regions of *LINC01443- LOC644669*, *TMTC1- IP O 8,* and *LINC02472- PCDH7*, show that the suggestive variants within these regions are in moderate-to-strong linkage disequilibrium (LD) with the sentinel variant (Supplementary Figures S2–S7). Variant-specific LD estimates generated from the GenHAT/REGARDS data are shown in Supplementary Table S6.

None of our single variant results were replicated in individuals from the WPC at a threshold of *p* < 5.00E-04 (*p* = 0.05/100 suggestive variants from Table 2 and Supplementary Table S3). Of the 10 statistically significant variants, rs144162260 in *COL12A1*, rs536017124 and rs192840029 in the *TMTC1-IPO8* intergenic region, and rs58633304 in the *LINC01443-LOC644669* intergenic region had the same direction of effect but non-significant *p*-values (Supplementary Table S3). Our extended look-up of variants ±100 kb of the target variant, identified seven intronic variants within the *NTM* gene that had *p*-values <5E-04. These variants were all located within 60 kb from either rs185159493 or rs184866696 (Supplementary Table S7).

Our lookup efforts in the COMPASS summary statistics provided marginal support for replication at the gene level, as none of the variants from our discovery were identified in COMPASS, most likely due to differences in imputation reference panels. Genes of interest based on the meta-analysis results included *COL12A1, NTM, PTPRR,* and *SNTG1*, as well as the *LINC01443*-*LOC64466, LINC02472*-*PCDH7,* and *TMTC1-IPO8* intergenic regions. While we could not replicate our meta-analysis findings on the single-variant level, rs192315401, an upstream variant of the *LINC02472*-*PCDH7* reached nominal significance in COMPASS (Supplementary Table S8, *p* = 3.20E-04). Results from the meta-analysis of MEGASTROKE top findings (*N* = 356 variants with *p* < 1.00E-07) with our AA data are presented in Supplementary Table S9. The top findings from MEGASTROKE were not significant in our data and the race-combined meta-analysis did not notably change any MEGASTROKE findings. We specifically focused on variants located within two well-characterized stroke loci: *PITX2* (*n* = 118) and *HDAC9* (*n* = 7). For the 118 *PITX2* variants, 102 had a consistent direction of effect across all three cohorts, while the remaining 16 were consistent across MEGASTROKE and REGARDS. In the *HDAC9* gene locus, all seven variants had the same direction of effect across all three cohorts (Supplementary Table S9).

We utilized FUMA to identify any tissue specificity of genes represented in our top findings. Of 64 unique gene names identified among variants with *p* < 1.00E-06, 57 were mapped to Ensembl identifiers by FUMA. Tissue analysis on an *a priori* selected 19 specific tissue types from GTEx revealed statistically significant, differential upregulation in brain tissues, specifically the hippocampus (p_adj_ = 9.67E-05), hypothalamus (p_adj_ = 2.05E-03), amygdala (p_adj_ = 5.28E-03), frontal cortex BA9 (p_adj_ = 6.36E-03), putamen basal ganglia (p_adj_ = 1.56E-02), anterior cingulate cortex BA24 (p_adj_ = 1.62E-02), and the caudate basal ganglia (p_adj_ = 3.17E-02) (Supplementary Table S10).

**FIGURE 2:**
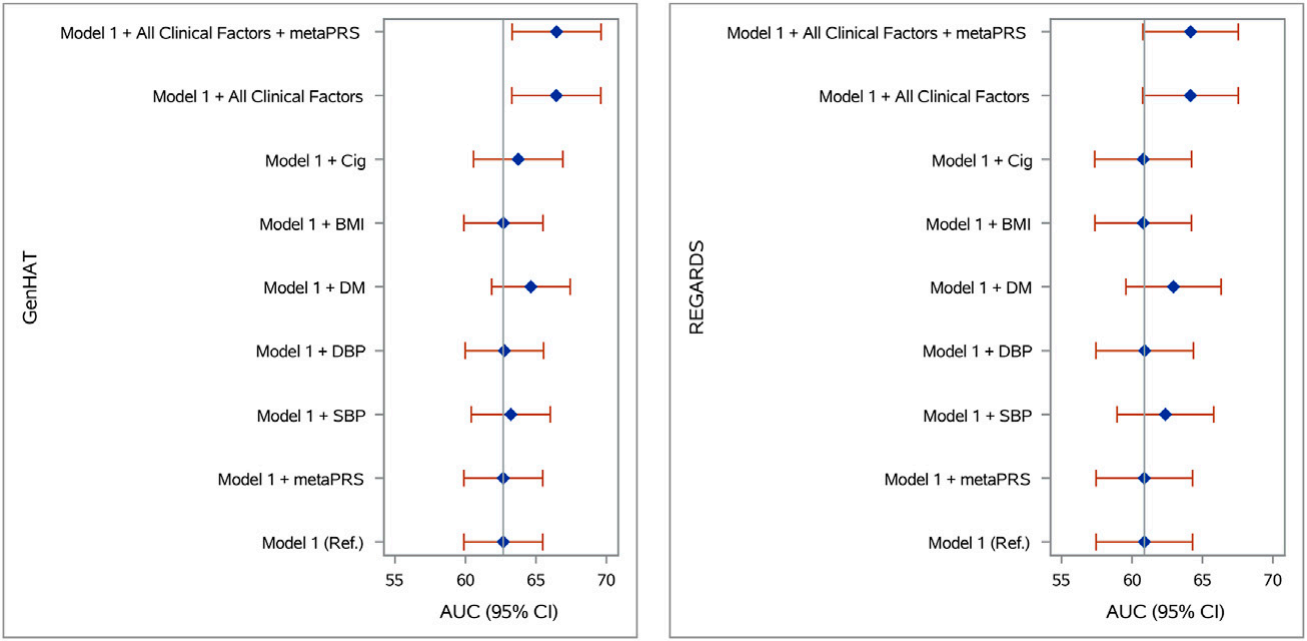
Stroke prediction model comparison in **(A)** GenHAT and **(B)** REGARDS populations. Shown are the area under the receiver operator characteristic curve (AUC) for eight logistic regression models (with and without the Abraham et al. metaPRS and clinical risk factors) used to predict stroke risk in comparison to a basic model 1 (age + sex +10 ancestry PCs). Error bars represent the 95% confidence intervals of the AUC. Abbreviations: PRS, polygenic risk score; Ref, reference model; SBP, systolic blood pressure; DBP, diastolic blood pressure; DM, diabetes mellitus; BMI, body mass index, kg/m^2^; Cig, cigarette smoking at baseline.

Accuracy in predicting incident stroke was assessed in GenHAT and REGARDS separately, using variant weights for over 400,000 overlapping variants in the UKB metaPRS (Abraham et al., 2019) (Supplementary Figure S8). In GenHAT, model 1 (AUC 62.68%; 95% CI 59.89–65.48%) was not statistically different than the model adding the metaPRS (Model 1 + metaPRS; AUC 62.68%; 95% CI 59.89–65.48%; *p* = 0.981). However, the models adding DM (Model 1 + DM; AUC 64.64%; 95% CI 61.68–67.43%; *p* = 0.025), all the clinical factors (Model 1 + All Clinical Factors; AUC 66.44%; 95% CI 63.29–69.60%; *p* = 0.003), and all the clinical factors plus the metaPRS (Model 1 + All Clinical Factors + metaPRS; AUC 66.46%; 95% CI 63.31–69.62; *p* = 0.003) were significantly different from the reference (i.e., Model one; Supplementary Table S11). Less than 0.01% of the variance was attributed to the metaPRS in GenHAT based on the difference of Nagelkerke pseudo-R^2^ values between the reference and metaPRS models (Figure 2; Supplementary Table S11). Similar results were observed in the REGARDS population. The reference model 1 (AUC 60.88%; 95% CI 57.45–64.30%) was not statistically different to the metaPRS model (AUC 60.87%; 95% CI 57.45–64.30%; *p* = 0.856). The models adding DM (AUC 62.94%; 95% CI 59.57–66.32%; *p* = 0.035), all the clinical factors (AUC 64.15%; 95% CI 60.76–67.54; *p* = 0.006), and all the clinical factors plus the metaPRS (AUC 64.16%; 95% CI 60.77–67.54; *p* = 0.006) were statistically significant, while the model adding SBP (AUC 62.37%; 95% CI 58.94–65.80%; *p* = 0.071) was marginally significant. Likewise, less than 0.001% of the variance was explained by adding the risk score for REGARDS based on the pseudo-R^2^ values between the reference and metaPRS model (Figure 2; SupplementaryTable S12).

## Discussion

While numerous stroke outcome GWAS have been published in the past several years, few studies have been performed exclusively in AAs. Using data from over 10,700 individuals from the GenHAT and REGARDS studies, we identified 10 statistically significant and an additional 90 suggestive genetic variants associated with incident stroke in individuals with HTN. While none of our findings were directly replicated, their gene level associations with stroke warrant future replication efforts, particularly variants located in or near the *NTM* or *PCDH7* genes.

In our meta-analysis, we identified 10 statistically significant variants, including six intergenic variants between a long-intergenic non-protein coding RNA (*LINC01443*) and a pseudogene (*LOC644669*). Four of these six variants were identified in a 2020 intracranial aneurysm (IA) GWAS in an East Asian population (Bakker et al., 2020). Additional significant findings include those variants located in *COL12A1.* This gene encodes the collagen alpha chain of type XII collagen, which interacts with type 1 collagen-containing fibrils (UniProt, 2021), and is a predicted target of the human microRNA-21, which is induced by Angiotensin II (Wang et al., 2017). Angiotensin II is the principal effector hormone of the renin-angiotensin system and increases blood pressure through vasoconstriction and increased sodium and water retention (de Leeuw, 1999), which is clinically relevant to our hypertensive study base.

Of additional interest were the variants downstream of the *TMTC1* (transmembrane O-Mannosyltransferase Targeting Cadherins 1) gene. *TMTC1* encodes a protein that transfers mannosyl residues to the hydroxyl group of serine or threonine residues and is primarily dedicated to the cadherin superfamily (UniProt, 2021). In a prior meta-analysis, variants in *TMTC1* were associated with heart failure (HF) in African ancestry populations (Smith et al., 2010). Other *TMTC1* variants have been associated with lipid metabolism (Talmud et al., 2009; Della-Morte et al., 2011). A 2011 study using data from the Northern Manhattan Stroke Study identified an interaction of *TMTC1* with abdominal obesity contributing to phenotypic variation in left ventricular mass (LVM). Increased LVM is a known risk factor for HF, stroke, and CVD (Della-Morte et al., 2011).

The only variants with gene-based replication were located in the first intron of *NTM* and upstream of *PCDH7. NTM* encodes a member of the IgLON glycosylphosphatidylinositol-anchored cell adhesion molecular family (Gene, 2004) and is primarily expressed in the heart and lungs (GTEx Consortium, 2015). A 2012 study found a balanced translocation break in *NTM* in a family with intracranial and thoracic aortic aneurysms (Luukkonen et al., 2012). Furthermore, intronic *NTM* variants have been previously implicated in intracerebral hemorrhage and small vessel ischemic stroke in Europeans (Chung et al., 2019). Additional studies have reported associations between *NTM* and CVD risk factors such as triglyceride levels (Li et al., 2015) and elevated protein levels in plasma serum (Cao et al., 2015). A recent study from the International Consortium for Antihypertensive Pharmacogenomics Studies concluded that variation in the *NTM* gene is associated with an increased risk of adverse cardiovascular outcomes in patients treated with beta-blockers, as well as an increase in blood pressure after beta-blocker treatment (McDonough et al., 2021). Although not statistically significant, we identified 11 variants located upstream of the *PCDH7* (protocadherin 7) gene. In previous studies, *PCDH7* was differentially expressed in aneurysm wall tissue compared to superficial temporal artery tissue (Shi et al., 2009), as well as being associated with white matter hyperintensities in EAs with ischemic stroke (Traylor et al., 2016).

Of the three marginally associated intronic variants within the *SNTG1* (syntrophin gamma-1) gene, rs117962542 has been previously implicated with stroke risk in a sample of ∼70,000 individuals of European descent from MEGASTROKE (Malik et al., 2018). *SNTG1* encodes a protein that mediates gamma-enolase trafficking to the plasma membrane and enhances its neurotrophic activity (UniProt, 2021). An epistasis analysis performed in 2,800 EAs found an association between an *SNTG1* variant and a history of arterial HTN (Zhou et al., 2020). Tissue specificity of our top identified genes, specifically *SNTG1* and *NTM*, showed differential expression in specific brain tissues, compared to other available tissues from GTEx RNA sequence data.

Complex diseases, such as stroke, have shown additive genetic architecture in previous association studies, making PRS a widely lucrative approach. PRS have been utilized to estimate an individual’s lifetime genetic risk of disease. While the current discriminative ability is low in the overall population, PRS may be useful in populations where there is a higher probability of disease to assist in prevention or diagnosis, or to inform treatment choices (Lewis and Vassos, 2020). Currently, there are several pitfalls of PRS implementation (Arnold and Koenig, 2021). One of the most impactful is the shortage of data describing PRS performance in African populations, especially since differences in allele frequencies and/or LD may limit cross-ethnic utility (Martin et al., 2019). Our data show that the application of the genome-wide Abraham metaPRS (derived and validated in >400K EAs) to >10,000 AAs does not aid in stroke prediction beyond age, sex, and genetic ancestry (reference model). We chose to test the Abraham metaPRS compared to other previously published PRS due to the metaPRS being similarly predictive to several stroke risk factors, including family history, BP, BMI, and smoking, as well as reports that the metaPRS (HR 1.26; 95% CI 1.22–1.31 per SD) doubled the predictive accuracy of ischemic stroke compared to the 90-variant score (HR 1.13, 95% CI 1.10–1.17) (Rutten-Jacobs et al., 2018; Abraham et al., 2019) in EAs. In both GenHAT and REGARDS, the models accounting for clinical risk factors (SBP, DBP, DM, BMI, and cigarette smoking) had the highest predictive accuracy and a negligible improvement was observed when adding the metaPRS. This suggests that Abraham score utility is not transferable to AA adults. Therefore, there remains a strong need for the generation and validation of stroke scores in minority populations, specifically AAs.

This study has several strengths. Both GenHAT and REGARDS collected adjudicated stroke data on a large sample of AAs with HTN who are disproportionally at-risk for stroke. We also utilized contemporary genotyping and imputation methods designed to be more inclusive for research in minority populations, allowing for more accurate genetic interrogation of our population (e.g., linkage patterns). We also must note some weaknesses. We were unable to focus on stroke type or sub-type due to a lack of that data in ALLHAT/GenHAT. Additionally, because of our inclusion/exclusion criteria focused on HTN, our findings may not be generalizable to younger, healthier populations, or individuals taking other antihypertensives (i.e., not a thiazide diuretic or ACE inhibitor). With our study base we may not find HTN genes related to stroke. However, this is an issue in older AA population studies of stroke in general as the prevalence of HTN is high (e.g., ∼70% in the parent REGARDS study). This could also be reflected in the FUMA results, where there was limited differential expression in vascular, non-brain tissues (e.g., artery, heart). Additionally, the GTEx data used in the FUMA analysis is comprises only 12.9% AA samples. Also, the lack of single-variant replication in the WPC was limited by the lower allele frequency variants from the discovery being underrepresented in the smaller sample size of the WPC. Finally, while the relatively recent and more racially inclusive TOPMed imputation panel allowed for interrogation of millions of novel variants, many of which were of lower allele frequency, it limited our replication efforts in published data that was imputed into earlier reference datasets, which highlights the necessity for additional stroke datasets in AAs.

In conclusion, we identified 10 statistically significant variants associated with incident stroke in AAs with HTN. The individual variants were not independently validated in the WPC or previously reported. However, many gene regions were biologically plausible and we found gene-based validation in previously published data. When accounting for case-control imbalance using SAIGE, our top findings remained significant and the same direction of the effect size was observed. This highlights the need for additional validation in large, stroke studies with AA populations and contemporary methods (e.g., use of whole genome sequence data and/or TOPMed imputed data) to capture genetic variation in non-European populations. The majority of the results identified genes (*NTM, PCDH7, COL12A1*) related to stroke or other CVD related diseases in Asian and European ancestral populations, with the exception of one region near *TMTC1*. This suggests both ancestry-common and ancestry-specific stroke risk genes are present in AAs. As hypothesized, this will necessitate discovery efforts to be more inclusive as genetic diagnostics trained on GWAS data are considered for use in the clinic. To that point, the published Abraham composite metaPRS trained in a large sample of EAs was not validated in the REGARDS or GenHAT study AAs. This research highlights the need to collect additional data through large biobanks and consortia efforts that can alleviate the potential for genetic discovery disparities.

## Data Availability

The raw GenHAT genotypic and phenotypic data used in this study are deposited in the National Center for Biotechnology Information (NCBI) Database for Genotypes and Phenotypes (dbGaP), accession number phs002716.v1.p1. The raw REGARDS genotype and phenotype data used in this study can be found in dbGaP, accession number phs002719.v1.p1.
